# On the Mixed Gas Behavior of Organosilica Membranes Fabricated by Plasma-Enhanced Chemical Vapor Deposition (PECVD)

**DOI:** 10.3390/membranes12100994

**Published:** 2022-10-13

**Authors:** Jens Rubner, Soukaina Skribbe, Hannah Roth, Lara Kleines, Rainer Dahlmann, Matthias Wessling

**Affiliations:** 1 Chemical Process Engineering AVT.CVT, RWTH Aachen University, Forckenbeckstraße 51, 52074 Aachen, Germany; 2 DWI—Leibniz-Institute for Interactive Materials, Forckenbeckstraße 50, 52074 Aachen, Germany; 3 Institute for Plastics Processing (IKV), RWTH Aachen University, Seffenter Weg 201, 52074 Aachen, Germany

**Keywords:** gas separation, mixed gas permeation, mixed gas selectivity, organosilica membrane, plasma-enhanced chemical vapor deposition

## Abstract

Selective, nanometer-thin organosilica layers created by plasma-enhanced chemical vapor deposition (PECVD) exhibit selective gas permeation behavior. Despite their promising pure gas performance, published data with regard to mixed gas behavior are still severely lacking. This study endeavors to close this gap by investigating the pure and mixed gas behavior depending on temperatures from 0 °C to 60 °C for four gases (helium, methane, carbon dioxide, and nitrogen) and water vapor. For the two permanent gases, helium and methane, the studied organosilica membrane shows a substantial increase in selectivity from α_He/CH_4__ = 9 at 0 °C to α_He/CH_4__ = 40 at 60 °C for pure as well as mixed gases with helium permeance of up to 300 GPU. In contrast, a condensable gas such as CO_2_ leads to a decrease in selectivity and an increase in permeance compared to its pure gas performance. When water vapor is present in the feed gas, the organosilica membrane shows even stronger deviations from pure gas behavior with a permeance loss of about 60 % accompanied by an increase in ideal selectivity α_He/CO_2__ from 8 to 13. All in all, the studied organosilica membrane shows very promising results for mixed gases. Especially for elevated temperatures, there is a high potential for separation by size exclusion.

## 1. Introduction

Since energy efficiency is crucial for mitigating global warming, industry is forced to develop alternative processes with reduced energy demand [1]. One promising alternative for gas separation is membrane processes, which are already used in different applications such as gas sweetening, hydrogen recovery and air separation [2]. These processes are up to 10 times more energy efficient than traditional separation methods [3]. As Sholl and Lively [3] state, the full potential for the use of gas separation membranes in chemical processes has not yet been reached. Membranes with high permeances and selectivities for specific applications are needed [3]. To fulfill the need for membranes with improved permeation performances, researchers have developed many new membrane materials in recent decades. Thereby, they were able to push the Robeson upper bounds for different gas combinations to higher values [4,5,6,7]. Although these new materials are promising, only a few materials developed decades ago are used for industrial membrane processes today due to the lack of scalable fabrication routes [8].

In this regard, a promising separation task for membranes is the recovery of helium. The demand and consequently the market prices of helium rose quickly in the last decades [9]. Helium is produced by separating it from natural gas fields with high helium contents (up to around 4%). While cryogenic distillation and pressure swing adsorption are commonly used recovery methods, in many cases, separation through membrane processes presents a more energy efficient alternative [9,10,11]. Sunarso et al. [12] and Scholes and Ghosh [13] reviewed different membrane materials for helium recovery in detail [12,13]. They found that especially silica membranes, in comparison to all other membrane materials, show high helium permeability and selectivity, which are essential for a successful implementation [12]. Although zeolites and metal organic frameworks (MOF) show similar permeabilities as silica, they suffer from low selectivity. Moreover, the loss in selectivity and also permeability significantly increases for MOF mixed-matrix membranes (MMM). On the one hand, the MMMs outperform polymeric membranes in permeability, selectivity and chemical stability. On the other hand, their production process is time consuming, hardly reproducible, difficult to upscale and hence costly. Thus, commercial membrane development for helium production still mainly focuses on polymeric membrane materials [8].

In addition to the previously mentioned materials, scalable plasma processes create thin selective coatings [14]. In particular, plasma-enhanced chemical vapor deposition (PECVD) has been studied for many years [15]. It allows the fabrication of tailored coatings with regard to mechanical and chemical properties and, at the same time, enables low coating thicknesses in the nanometer range. The formation of organosilica layers by PECVD utilizing hexamethyldisiloxane (HMDSO) as a precursor results in membranes with gas separation characteristics [15,16,17,18,19,20,21,22,23]. Inorganic [24,25,26,27] or organic substrate membranes [15,21,22,23,28,29,30,31,32,33] function as support for the thin organosilica layer. Besides, PECVD enables the combination of advantages of different membrane materials. Inexpensive organic membranes offering mechanical support combined with an organosilica layer result in high permeances and selectivities for helium [26]. Furthermore, PECVD is already industrially used and scalable in roll-to-roll processes and therefore can be easily applied for industrial scale membrane production [34]. Since the resulting selective layer properties are dependent on the plasma parameters [22,23], membranes for different separation tasks can be fabricated on the same production line by only changing the plasma parameters. While PECVD membranes are not a new concept in the literature of the last two decades, research does not offer much information on mixed gas experiments with such membranes. The interaction effects of different gases and vapors on the permeance and selectivity of those membranes are still unknown.

**Figure 1:**
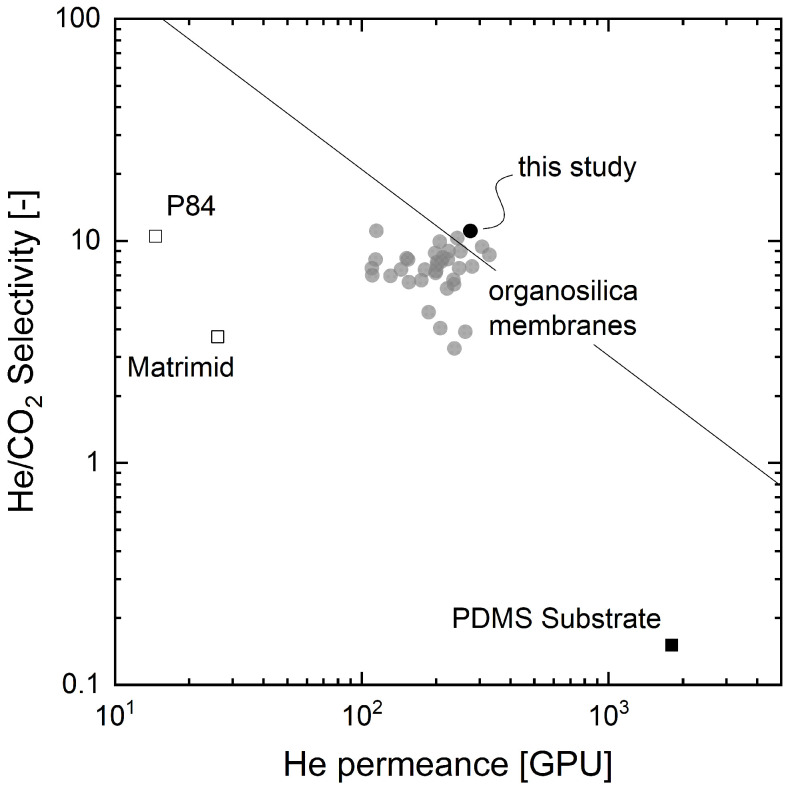
Robeson plot for He/CO_2_ with a variety of organosilica membranes fabricated via PECVD by the authors (filled circles) [21,22] and the uncoated PDMS substrate membrane. As reference, the plot displays two commercially available polymers (Matrimid [36] and P84 [37]) with an assumed thickness of 1 µm. The best performing organosilica membrane, which was chosen for mixed gas experiments in this study, is plotted with a black circle.

To gain more knowledge about the permeation behavior of membranes fabricated by PECVD, this study closely investigates the performance of one promising organosilica membrane. Figure 1 shows the selectivity of helium to carbon dioxide over the helium permeance in an adapted Robeson plot. Since there is no clear linear relation between permeance and thickness in organosilica membranes fabricated by PECVD, unlike what is generally seen in conventional polymers, a thickness of 1 µm is assumed for all polymer membranes, according to [35]. The solid line in Figure 1 represents the Robeson upper bound [4]. The filled circles show the permeation characteristics of our composite membranes with selective PECVD coatings fabricated with varying coating parameters (refer to Kleines et al. [21,22]). Additionally, the characteristics of the used PDMS substrate for the PECVD coating and two commercially available polymers (Matrimid [36] and P84 [37]) are plotted as reference.

For this study, the best-performing PECVD membrane, plotted as a black circle (cf. Figure 1), was chosen for further investigation. First, this study determines the activation energies for pure gas permeation of four gases (He, CO_2_, CH_4_, and N_2_) from 0 °C to 60 °C. Then, it evaluates the activation energy differences of the four gases and compares them to published PECVD membrane data, which all show high energy values [18,24,26,27,38]. Afterward, the study focuses on the mixed gas behavior of equimolar mixtures of selected gases. Experiments at various temperatures will elucidate the possible occurrence of competitive sorption or other non-ideal effects of organosilica membranes. In the last step, the study assesses the influence of additional water vapor on the pure gas permeance of the most promising gas combination, He and CO_2_. It further discusses the possibility of water vapor condensation or organosilica hydrolysis, previously reported for PECVD membranes [39,40,41,42,43,44]. This study aims to identify application windows for gas separation tasks with our scalable producible organosilica membrane.

## 2. Experimental

### 2.1. Materials

A PDMS composite membrane acts as the substrate for the deposition of the PECVD layer and was supplied by the Helmholtz-Zentrum Hereon Geesthacht. It consists of a non-woven fabric, a microporous support structure of polyacrylonitrile (PAN) and a dense, about 160 nm-thin, gas-selective layer of polydimethylsiloxane (PDMS). This substrate membrane was chosen because of its smooth surface compared to porous substrates, which should enable a more homogeneous, uniform, and defect-free layer growth. Furthermore, PDMS is a rubbery polymer and therefore known for its high permeance and its nearly ideal permeation behavior.

**Figure 2:**
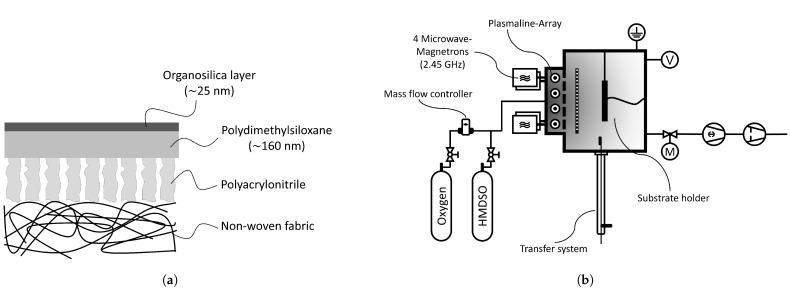
Schematic representation of (**a**) the membrane’s multilayer structure and (**b**) the low-pressure plasma reactor LAMPS.

For the PECVD coating, this study uses hexamethyldisiloxane (HMDSO) as a monomer and oxygen as an auxiliary reaction gas for the deposition of the plasma polymer layers. HMDSO with a purity of >98% was purchased from Sigma-Aldrich. Figure 2a shows the schematic layer structure of the final membrane. For the permeation experiments, helium (He) and methane (CH_4_) from Nippon with a purity of 99.999 vol.-% and 99.95 vol.-%, respectively, were used. Carbon dioxide (CO_2_) with a purity of 99.995 vol.-%, and nitrogen (N_2_) from Westfalen AG with a purity of 99.999 vol.-% were used.

### 2.2. PECVD Membrane Fabrication

The coating deposition was conducted in a low-pressure plasma reactor (LAMPS, large-area microwave plasma system; see Figure 2b). Four microwave magnetrons generate pulsed microwaves with a frequency of 2.45 GHz. The microwaves are fed into the reaction chamber via four quartz glass tubes (Duo-Plasmalines, Muegge GmbH, Reichelsheim). Detailed information about the reactor design can be found elsewhere [45].

A 7 × 7 cm piece of the PDMS composite membrane mentioned in Section 2.1 is installed on the substrate holder of the LAMPS to deposit the organosilica membrane on top. First, an oxygen plasma pre-treatment activates the surface before coating (see Table 1 for plasma parameters) [22,23]. Afterward, the organosilica layer is deposited using HMDSO as a precursor in the pulsed plasma with a microwave peak power of 2000 W for 93 s. An organosilica layer with a thickness of about 25 nm is deposited. At the same time, a silicon wafer and a gold-sputtered silicon wafer are coated next to the membrane for subsequent ellipsometry and FTIR measurements, respectively.

### 2.3. XPS Measurements

The X-ray photoelectron spectroscopy (XPS) measurements were carried out in an Ultra Axis spectrometer from Kratos Analytical (Manchester, UK). The membrane samples were irradiated with mono-energetic Al K*1,2 radiation (1486.6 eV), and the spectra were taken at a power of 144 W (12 kV × 12 mA). The aliphatic carbon at a binding energy of 285 eV (C 1s photo line) was used to determine the charging. XPS spectra were processed with dedicated software, and atomic concentrations of the elements were quantified by integration of the relevant photoelectron peaks. The information depth was about 10 nm.

### 2.4. FTIR Measurements

Structural chemical properties were investigated by Fourier transform infrared spectrometry (FTIR) with a Nexus 870 spectrometer from Thermo Nicolet (now Thermo Fisher Scientific). The infrared spectrometer was operated in attenuated total reflection (ATR) mode with a diamond crystal at a fixed angle of 42° or 45° and a potassium bromide beam splitter. Gold-sputtered silicon wafers served as substrate for the PDMS and PECVD layer.

### 2.5. Ellipsometry Measurements

To determine the thickness of the deposited PECVD layer, this study conducted ellipsometric measurements with a spectroscopic ellipsometer (RC2 from J.A. Woollam Co., Inc., Lincoln, NE, USA) with a investigated spot size of 2 mm. All measurements were performed at incident angles of 65°, 70° and 75° over a wavelength range of 300 to 1000 nm with a spectral resolution of 2 nm. The optical modeling was done with the commercial software package CompleteEASE 6.46 (J.A. Woollam Co.).

The layer thickness was measured on a silicon wafer and not on the membrane itself to avoid thickness deviations originating from the slightly varying PDMS layer thickness of the membrane. Material data for silicon were taken from Herzinger et al. [46]. To determine the PECVD layer thickness, a Cauchy-type parametrization ($n\left(\lambda \right)=A+B/\lambda^{2}$) was used and fitted to the data. The determined thickness of the deposited layer is stated in Table 1.

### 2.6. Permeation Measurements

**Figure 3:**
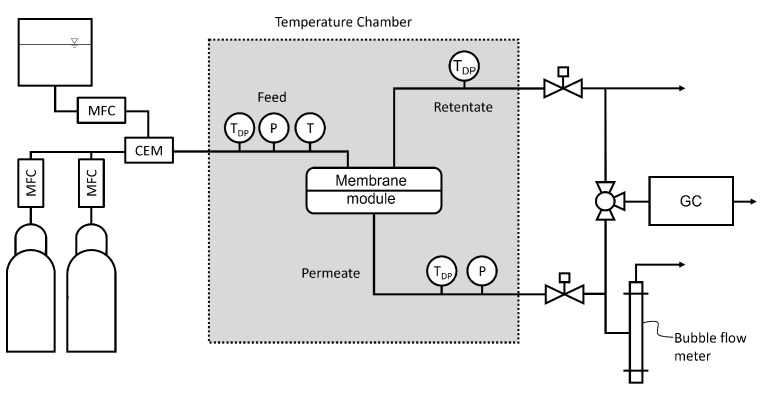
Simplified flowsheet of the gas permeation setup for pure gas, mixed gas, and pure gas with water vapor experiments. The setup consists of mass flow controllers for gases and water (MFC), a controlled evaporator and mixing device (CEM), dew point (T_DP_), temperature (T), and pressure (P) sensors and is connected to a gas chromatograph (GC).

This study conducted all permeation measurements with a constant-pressure/variable volume method on a gas permeation setup (GPS) similar to the one used by Logemann et al. [47]. Figure 3 shows a simplified flowsheet of the GPS. The membrane module is located in an air-conditioned cabinet (Memmert GmbH), which enables experiments in the temperature range from −20 °C to 60 °C in this study. The diameter of the membrane is 65 mm. The feed pressure is regulated with an automated back pressure regulator (Bürkert GmbH) and set to 2 bara for all experiments. The permeate pressure is always ambient pressure (1 bara).

The gas mixtures were produced from pure gas cylinders with the required composition controlled through mass flow controllers (Bronkhorst EL-Flow). To determine the gas compositions of the feed, retentate and permeate stream, a gas chromatograph (Agilent Technologies 7890A) was connected to the gas permeation setup, which consists of a thermal conductivity detector and argon as carrier gas. For experiments with water vapor, a controlled evaporation and mixing device (CEM Evaporator W-101A from Bronkhorst GmbH) was used. The water vapor content was measured using dew point mirrors (Michell Optidew) in the feed, retentate and permeate channels. The measured dew points were converted into water vapor pressures using the Antoine equation [48]. With this value, the water activity $a_{w}$ is calculated as follows:

(1) $$a_{w}=\frac{p_{H2O,gas}}{p_{H2O,sat}\left(T\right)},$$

where $p_{H2O,gas}$ is the water vapor pressure of the gas stream measured with the dew point sensor, and $p_{H2O,max}\left(T\right)$ is the saturation water vapor pressure at the temperature of the gas stream.

A manual bubble flow meter was used to determine the permeate flux. The permeances are calculated with:

(2) $$Q_{i}=\frac{{\dot{V}}_{P}x_{P_{i}}\left(\frac{T_{F}}{273.15+T_{R}}\right)}{A_{m}\left(p_{F_{i}}-p_{P_{i}}\right)},$$

where $Q_{i}$ is the permeance (GPU) (1 GPU = 10^−6^ cm^3^(STP)/cm^2^· s· cmHg), ${\dot{V}}_{P}$ is the average permeate volume flux, $x_{P_{i}}$ the mole fraction of gas i in the permeate stream, $T_{F}$ is the feed temperature, $T_{R}$ is the room temperature, $A_{m}$ is the membrane surface area, and $\left(p_{F_{i}}-p_{P_{i}}\right)$ is the partial pressure difference between the feed and the permeate side.

The apparent activation energies of permeation for the gases i, $E_{P,i}$, were calculated using the Arrhenius-type equation:

(3) $$Q_{i}\left(T\right)=Q_{0,i}\cdot \exp\frac{-E_{P}}{RT},$$

where $Q_{i}\left(T\right)$ is the permeance of gas i at temperature *T*, $P_{0,i}$ a pre-exponential factor for gas i, *R* is the ideal gas constant, and *T* is the absolute temperature.

## 3. Results and Discussion

### 3.1. Chemical Analysis of the Organosilica Layer

The elemental composition of the PDMS substrate membrane and the organosilica layer fabricated by PECVD is determined by XPS at three different spots of the membrane. Table 2 displays the elemental composition of the PDMS and the organosilica layer. Each has a thickness significantly higher than 10 nm to avoid influences of the underlying PAN and PDMS layer, respectively. Since the XPS can not detect hydrogen atoms, the content of this atom is disregarded in Table 2 and the following paragraph.

The measured atomic composition of the investigated PDMS membrane layer (SiO_1.1_C_2.3_) is in accordance with its theoretical composition SiOC_2_. Considering the organosilica layer, the atomic composition of it (SiO_1.1_C_1.9_) only deviates marginally from the theoretical PDMS and the measured PDMS membrane composition. Compared to the used monomer HMDSO (SiO_0.5_C_3_), the deposited organosilica layer shows a significantly lower carbon and a higher oxygen amount. The change in carbon content can be attributed to a depletion of the methyl groups in the PECVD process. In contrast, the increase in oxygen content cannot be induced by the PECVD process, since there is no additional oxygen present. Rather, it could be attributed to the contact with atmospheric oxygen or water vapor after the coating process, possibly leading to slow post-oxidation promoted by free radicals incorporated in the organosilica layer during film growth [49].

**Figure 4:**
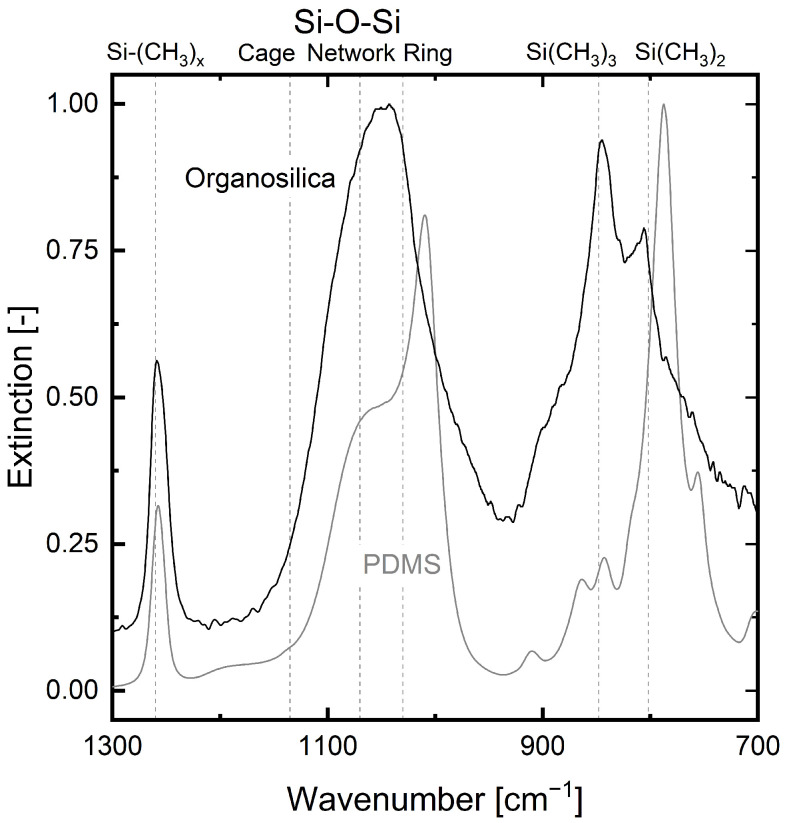
Infrared spectra from 1300 to 700 cm^−1^ of the PDMS (grey) and the PECVD-deposited organosilica layer (black). Wave numbers of the organosilica’s characteristic functional groups are marked by dashed lines [50,51,52].

Figure 4 displays the FTIR spectra from 1300 to 700 cm^−1^ of the PDMS and the PECVD-deposited organosilica layer. To allow comparability, the main peak of the PDMS and the organosilica spectra at about 1040 and 790 cm^−1^, are normalized to a value of 1. The characteristic wave numbers of the functional groups of organosilica layers are marked in Figure 4 for easier assessment [50,51,52].

The infrared (IR) spectrum of the PDMS coating exhibits characteristic IR bands [53,54]. The absorption band at around 1010 cm^−1^ can be attributed to the -Si-O-Si- group of the PDMS. The absorption bands at around 790 and 1270 cm^−1^ represent the valence vibrations of the -Si-C- and -C-H groups, respectively [54].

The IR spectrum of the organosilica layer has an absorption band at around 1270 cm^−1^ as well. However, in contrast to the PDMS, the organosilica layer has its main peak at around 1040 cm^−1^, correlating with the Si-O-Si vibration mode [50]. Figure 4 also shows absorption peaks at 845 and 805 cm^−1^, representing the methyl group contribution. These results are in accordance with measurements for pure HMDSO PECVD-fabricated layers published by Kleines et al. [22].

Together with the carbon content of nearly 50 % measured with XPS, this result confirms the strong organic character of the studied organosilica layer.

### 3.2. Temperature-Dependent Permeation Behavior

**Figure 5:**
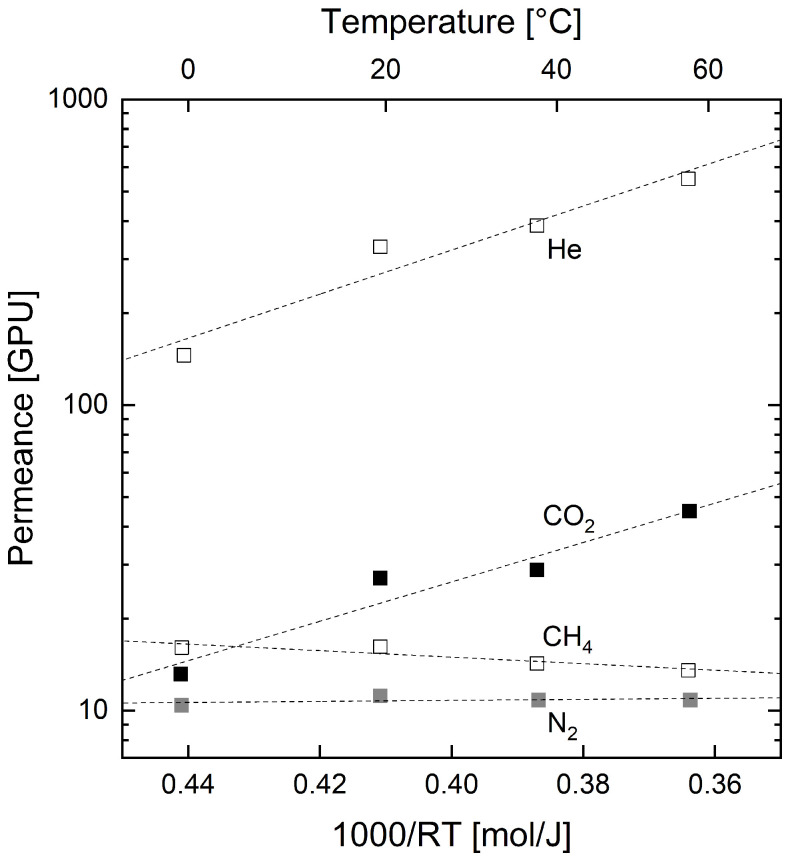
Permeances for He, CO_2_, CH_4_ and N_2_ of the studied organosilica membrane fabricated by PECVD. Permeances are plotted over 1000/(RT) to determine the activation energies for permeation for the different gases.

The pure gas permeance of the organosilica membrane was measured for He, CO_2_, CH_4_ and N_2_ at four different temperatures (0, 20, 40, 60 °C) and is depicted in Figure 5. The individual permeances are plotted over 1000/(RT). The dashed lines show the best fit for each gas, which represent an Arrhenius-type function. This enables the determination of the activation energy for permeation according to Equation (3).

He and CO_2_ show a clear increase in permeance over time, and their activation energies are 16.6±3.1 kJ/mol and 14.8±2.8 kJ/mol, respectively. In comparison, the activation energies for N_2_ and CH_4_, being 0.4±0.5 kJ/mol and −2.5±0.8 kJ/mol, respectively, are significantly lower. This distinct temperature-dependent behavior for He accompanied with a low or even negative activation energy for N_2_ compares well to other publications of membranes fabricated by PECVD [18,24,26,27,38]. Those substantial differences in activation energies are due to the microporous structure of the organosilica layer. The molecules with small kinetic diameters (He and CO_2_) are small enough to permeate through the micropores/free-volumes of the membrane following the solution-diffusion mechanism. At the same time, the bigger molecules cannot access those micropores but only permeate through defects (meso- and macropores) in the organosilica layer following Knudsen diffusion. Whilst Knudsen diffusion is only weakly temperature dependent, a temperature increase leads to a substantially increased diffusivity and hence also permeation for gases following the solution-diffusion behavior [20,24]. This permeation behavior leads to a remarkably strong selectivity increase with increasing temperature for CO_2_ to CH_4_, which has, to the best of our knowledge, not been reported for any gas separation membrane before. Furthermore, the differences in activation energies for the investigated gases lead to the assumption that the average pore/free-volume size of the studied organosilica layer is between the kinetic diameter of CO_2_ (330 pm [55]) and N_2_ (364 pm [56]) [20,24].

### 3.3. Mixed Gas Behavior

Non-ideal effects can potentially occur and drastically alter the separation performance of the membrane. The following section evaluates the gas pairs He/CH_4_, He/CO_2_ and CO_2_/CH_4_ with regard to their mixed gas behavior in comparison to the aforementioned pure gas experiments.

#### 3.3.1. Permanent Gases

**Figure 6:**
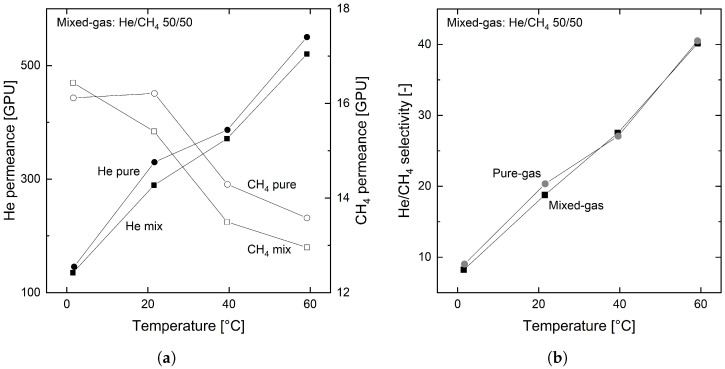
Permeance (**a**) and selectivity (**b**) of the organosilica membrane for He and CH_4_, pure gas (circles) and mixed gas (squares) experiments with feed and permeate absolute pressures of 2 and 1 bar, respectively.

First, the mixed gas behavior of the two permanent gases, He and CH_4_, is discussed. Figure 6a,b shows the permeances and the resulting selectivity for the pure and mixed gas experiments from 0 to 60 °C.

For mixed gases, the permeance of both gases drops slightly in comparison to the pure gas results. Nevertheless, the selectivity in the mixed gas stays the same as for the pure gas experiments. Hence, the organosilica membrane shows no significant non-ideal behavior in the investigated temperature and pressure range for permanent gases. The same behavior can also be expected for the mixture of the two permanent gases He and N_2_, which was not investigated in this study.

#### 3.3.2. Condensable Gases

Permeation experiments with CO_2_ reveal the influence of a condensable gas on the permeation behavior of the organosilica membrane. This section shows the results of the addition of CO_2_ to both He and CH_4_.

**Figure 7:**
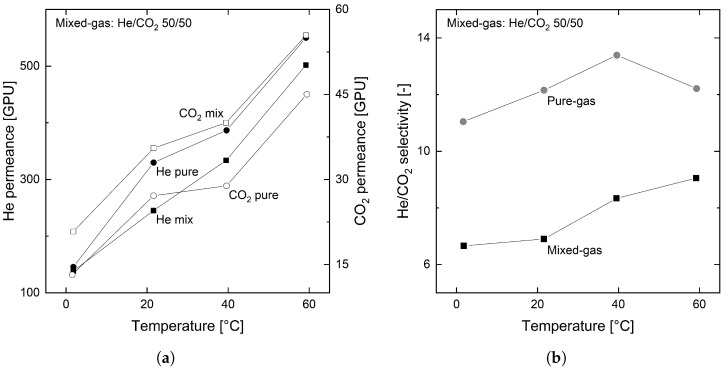
Permeance (**a**) and selectivity (**b**) of pure and mixed gas (50:50) He and CO_2_ for the organosilica membrane for 0, 20, 40 and 60 °C with feed and permeate pressures of 2 and 1 bar, respectively.

Figure 7a,b illustrate the permeances and selectivities of the pure and mixed gas experiments with He and CO_2_. In these experiments, the condensable CO_2_ is the retained compound.

As can be seen, the temperature-dependent behavior of both gases is the same for pure and mixed gas experiments. However, Figure 7a also shows a decrease in mixed gas permeance for He and an increase for CO_2_ compared to their pure gas values. This effect results in a significantly decreased mixed gas selectivity of He to CO_2_ (cf. Figure 7b). This behavior was also observed in other studies and is attributed to effects of plasticization and/or competitive sorption [57,58,59,60]. However, as Genduso et al. [61] recently showed, in case of 6FDA-mPDA, the depression of the size sieving capability of the polymer causes this decrease in selectivity. Hence, to elucidate the origin of this phenomenon, further studies on the sorption and diffusion behavior of the organosilica membrane need to be conducted.

Regardless of the selectivity loss for mixed gases, the trend of an increasing permeance and selectivity over temperature is maintained for the investigated gas mixture. Thus, this organosilica membrane favors the application at elevated temperatures.

**Figure 8:**
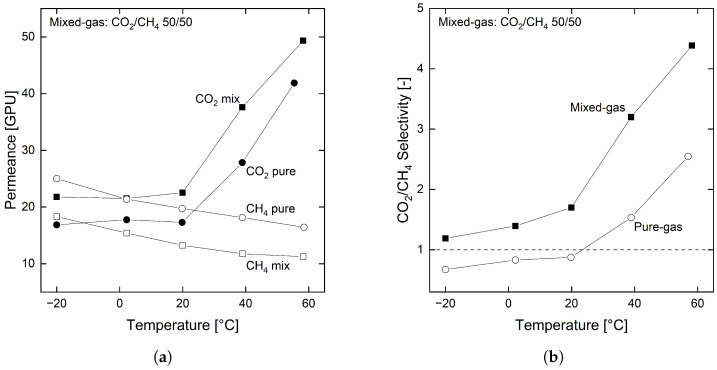
Permeability (**a**) and selectivity (**b**) of pure and mixed gas (50:50) CO_2_ and CH_4_ for the organosilica membrane. Results are plotted for −20, 0, 20, 40 and 60 °C with feed and permeate pressures of 2 and 1 bar, respectively.

Besides the mixed gas He and CO_2_, a 50:50 mixture of CH_4_ and CO_2_ also functions as feed. Figure 8 displays the permeances and selectivities for pure and mixed gas experiments of CO_2_ and CH_4_ from −20 to 60 °C.

A unique feature of the organosilica membrane is the inversion of selectivity for CH_4_ and CO_2_ pure gases with respect to temperature (see Figure 8b). This effect is based on the strongly differing activation energies for both gases (see Section 3.2). The permeance of CO_2_ strongly increases with temperature, whereas the permeance of CH_4_ slightly drops. This behavior leads to an inversion of selectivity between 20 and 40 °C in the pure gas experiment. In contrast, the CO_2_/CH_4_ selectivity for the mixed gas experiments does not show this inversion of selectivity but always remains above one (see Figure 8b), even for temperatures as low as −20 °C.

Figure 8a shows the course of the permeances of the two gases. Here, the mixed gas permeances of CO_2_ surpass the values of the pure gas experiments, whereas the permeances for CH_4_ lay below their pure gas values. This mixed gas effect can be attributed to the above mentioned competitive sorption effect [60,62]. As with He and CO_2_, the condensable CO_2_ apparently sorbs preferentially into the membrane, thereby mitigating the sorption of CH_4_. In concrete terms, this leads to an in- and decrease of the CO_2_ and CH_4_ permeance, respectively, preventing the inversion of selectivity. Further studies on the sorption and diffusion behavior of the organosilica layer are needed to gain more insights into the exact phenomena.

#### 3.3.3. Water Vapor

**Figure 9:**
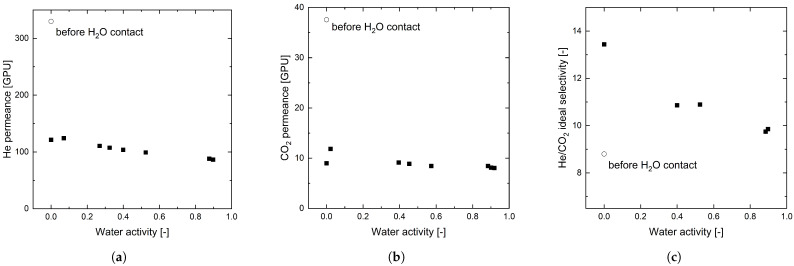
He permeance (**a**), CO_2_ permeance (**b**), and ideal He/CO_2_ selectivity (**c**) in dependence of water activity in the feed gas stream of the gas mixture. Values of the membrane before contact with H_2_O vapor are displayed with open symbols as reference.

Figure 9a,b shows the permeance of pure He and CO_2_ in dependence on the feed gas water activity in the respective gas at 25 °C. Before the membrane came into contact with water vapor, the He and CO_2_ permeances were at 330 and 37.5 GPU, respectively. As soon as water vapor contacts the membrane, the permeance drops by about 60 % to around 100 and 10 GPU, respectively, for low-water activities. When going back to pure gases, the permeance does not increase back to the original value. Even an extensive drying time (50 h) under vacuum and elevated temperatures (60 °C) does not regain the original value (data not shown).

At the same time, Figure 9c shows an increase in ideal selectivity for He to CO_2_ after contact with water vapor. Starting at a value of 8.8, the selectivity increases to 10 for high water activity and even reaches a value of more than 13 for dry gases after the organosilica membrane has been in contact with water vapor.

Various authors also reported a decreasing permeance when contacting (organo)silica membranes with water vapor [39,40,41,42,43,44,63]. Gavalas et al. [39] and Gallaher and Liu [40] attributed the permeance drop to moist air adsorbed in the pores and reversed this effect by heating above 600 °C [39,40]. Since the support membrane of this study is not stable in those conditions, only a temperature of 60 °C under vacuum was used, but a reversal of the drop in permeance could not be achieved.

However, Leboda et al. [41], Duke et al. [42], Castricum et al. [43] and Song et al. [44] attribute the change in permeance to hydrothermal instability of the silica membrane. The membranes lack inherent microstructural stability, and hence, exposure to water leads to hydrolysis of siloxane groups on the surface. Thereby, the mobile groups coming from bigger pores condense in small pores, which leads to pore closure. In the end, this effect leads to an increased permeance and decreased selectivity of the silica membranes [41,42,43,44].

In contrast, the membrane in this work shows the opposite behavior compared to the latter and hence seems to not be affected by hydrolysis. This is also in agreement with the findings of Duke et al. [42]. There, organic methyl groups, which are also present in our organosilica membranes, led to a stabilization of the membrane. Comparable to our findings, they still discovered a drop in permeance but observed a stable selectivity [42].

Nevertheless, we cannot completely rule out the possible presence of condensed water vapor in our organosilica membranes. To obtain a better understanding of the aforementioned behavior, detailed investigations should be carried out following this work.

**Figure 10:**
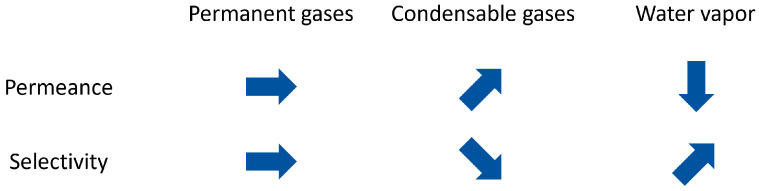
Effect of mixed gas on the separation behavior of organosilica membranes.

In summary, Figure 10 schematically shows all effects on permeance and selectivity induced by mixed gas compared to the pure gas results for the organosilica membrane. For permanent gases, this study did not see any mixed gas effects. In contrast, the condensable CO_2_ leads to an increase in permeance and a selectivity loss for mixed gases. Conversely, the addition of water vapor induces a significant drop in gas permeance together with increased selectivity.

## 4. Conclusions

Organosilica membranes fabricated via PECVD have the potential to combine the advantages of silica and polymeric membranes. Thereby, they offer both high permeabilities and selectivities similar to silica membranes and low production costs compared to polymeric membranes. Our in-depth investigation of a selected organosilica membrane with pure and mixed gas feeds at different temperatures reveals promising non-ideal permeation behavior.

For pure gases, He and CO_2_ permeance for the organosilica membrane are strongly temperature-dependent. In contrast, N_2_ and CH_4_ permeances stay constant or even decrease slightly with increasing temperature. This leads to a strong increase in ideal selectivity with increasing temperature. The studied organosilica membrane, for example, shows a substantial increase in selectivity from α_He/CH_4__= 9 at 0 °C to α_He/CH_4__= 40 at 60 °C, accompanied by a He permeance of up to 300 GPU.

Furthermore, mixed gas permeation effects with equimolar mixtures of He/CH_4_, He/CO_2_ and CO_2_/CH_4_ were studied. For the combination of two permanent gases (He and CH_4_), only minor changes in the permeance and a mixed gas selectivity equal to the ideal selectivity were observed. However, when mixing a permanent and condensable gas, the membrane exhibits a significantly decreased selectivity compared to ideal conditions, which we attribute to competitive sorption. The same phenomenon also seems to affect the CO_2_/CH_4_ selectivity. While the pure gas experiments show an inversion of selectivity over temperature, this behavior was not apparent for mixed gas. A selectivity above 1 for CO_2_/CH_4_ occurs over the investigated temperature range of −20 to 60 °C.

Furthermore, water vapor induces a strong effect on the permeances of He and CO_2_. A significant, irreversible drop in permeance appeared for both gases. However, at the same time, the organosilica membrane shows an increase in the ideal He/CO_2_ selectivity. The reason for this behavior might be water condensation in the organosilica membrane, but we can exclude hydrolysis of siloxane groups in the membrane.

All in all, the investigated organosilica membrane shows a promising mixed gas behavior. For the separation of helium or similar permanent gases with small kinetic diameters from other bigger gas molecules, we identified a promising operational window at elevated temperatures.

Nevertheless, the selectivities and permeances at ambient conditions still need to be improved to outperform known membranes under those conditions. Hence, further research on the fabrication parameters of PECVD membranes is necessary. Here, in particular, a deeper understanding of the PECVD process is needed. The ability to fabricate coatings with narrower free volume size distributions and to adjust the free volume size would allow the fabrication of tailored selective layers for specific separation tasks by size sieving. In continuation of this work, the assessment of the interaction of water vapor and other easily condensable components such as higher hydrocarbons with the organosilica membrane has to be studied in more detail to fully understand short and long-term effects on the membrane performance.

## Figures and Tables

**Table 1:** PECVD coating parameters.

Coating Type	HMDSO Flow [sccm]	Oxygen Flow [sccm]	Pulse on/off [ms]	Microwave Peak Power [W]	Coating Time [ms]	Thickness [nm]
Pre-Treatment	0	100	4/45	4000	5000	-
Layer Deposition	70	0	2/45	2000	93,000	∼25

**Table 2:** Elemental composition of the PECVD layer and PDMS substrate in at. % by XPS.

Layer	Si2p	O1s	C1s	Composition
PDMS	22.51 ± 0.18	25.11 ± 0.24	52.39 ± 0.38	SiO_1.1_C_2.3_
Organosilica	24.95 ± 0.70	27.38 ± 0.13	47.67 ± 0.57	SiO_1.1_C_1.9_

## Data Availability

The data presented in this study are available on request from the corresponding author.

## Abbreviations

The following abbreviations are used in this manuscript:

α_i/j_	Membrane selectivity of gas i to gas j
CEM	Controlled evaporator and mixing device
CH_4_	Methane
CO_2_	Carbon dioxide
GC	Gas chromatograph
GPS	Gas permeation setup
GPU	Gas permeation unit
He	Helium
HMDSO	Hexamethyldisiloxane
MFC	Mass flow controller
MOF	Metal organic framework
N_2_	Nitrogen
PAN	Polyacrylonitrile
PDMS	Polydimethylsiloxane
PECVD	Plasma-enhanced chemical vapor deposition
T	Temperature (°C)
T_DP_	dew point temperature (°C)
P	Pressure (bar)
XPS	X-ray photoelectron spectroscopy
